# Highlight: The Evolutionary Fates of Supergenes Unmasked

**DOI:** 10.1093/gbe/evab079

**Published:** 2021-05-04

**Authors:** Casey McGrath

Although the term “supergene” may bring to mind the genetic hocus-pocus of Peter Parker’s transformation into Spiderman, supergenes are actually fairly common phenomena in the realm of biology. A supergene refers to a genomic region containing multiple genes or genetic elements that are tightly linked, allowing genetic variants across the region to be co-inherited. Supergenes may arise when there is a clear benefit to inheriting specific combinations of biological traits together. Perhaps the most well-known examples of supergenes are sex chromosomes, which allow traits that are beneficial to the reproductive success of one sex to be co-inherited. In humans, this explains the prevalence of male-specific genes on the Y chromosome. Although the concept of supergenes arose nearly a century ago, until recently, the study of their origin, evolution, and eventual fate was largely theoretical. Now, however, thanks to advances in genomic sequencing and computational biology, scientists can put those theories to the test with real-world data. In a recent review published in *Genome Biology and Evolution* titled “The genomic architecture and evolutionary fates of supergenes,” Associate Professor Tanja Slotte and her colleagues at Stockholm University in Sweden discuss new findings in the field of supergene evolution and reveal how the genomic architecture of a supergene is inextricably tied to its evolutionary fate (Gutiérrez-Valencia et al. 2021). 

“There is a rich history in evolutionary biology when it comes to the study of supergenes,” says Slotte. “What I like about this topic is that there are theoretical models of supergene evolution to draw on, and at the same time, we can now thoroughly test those expectations empirically using genomic data or explore the expected effects of different genomic architectures using simulations.” In particular, these new approaches can be used to assess the validity of some of the more well-established theories about supergenes.

Classical models posit that supergenes arise following the establishment of mutations at a minimum of two sites, followed by sequential accumulation of additional mutations. Selection for specific combinations of variants acts to suppress recombination, thus strengthening the linkage between mutations. This suppression of recombination often occurs through inversions, in which a genomic region is flipped on the chromosome, effectively inhibiting recombination. Because recombination enables the removal of deleterious mutations, however, once it has stopped, the supergene may degenerate through the accumulation of single-nucleotide mutations, as well as insertions, deletions, and the replication of transposable elements. Notably, this last characteristic of supergenes has made them particularly difficult to study until now. Notes Slotte, “This is a very good time for studying supergene evolution. Thanks to long-read sequencing and improved bioinformatic methods, we can now obtain high-quality assemblies, including well assembled supergenic regions. This is not a trivial task, as non-recombining regions are often highly repetitive and therefore difficult to assemble.”

**Fig. 1.**
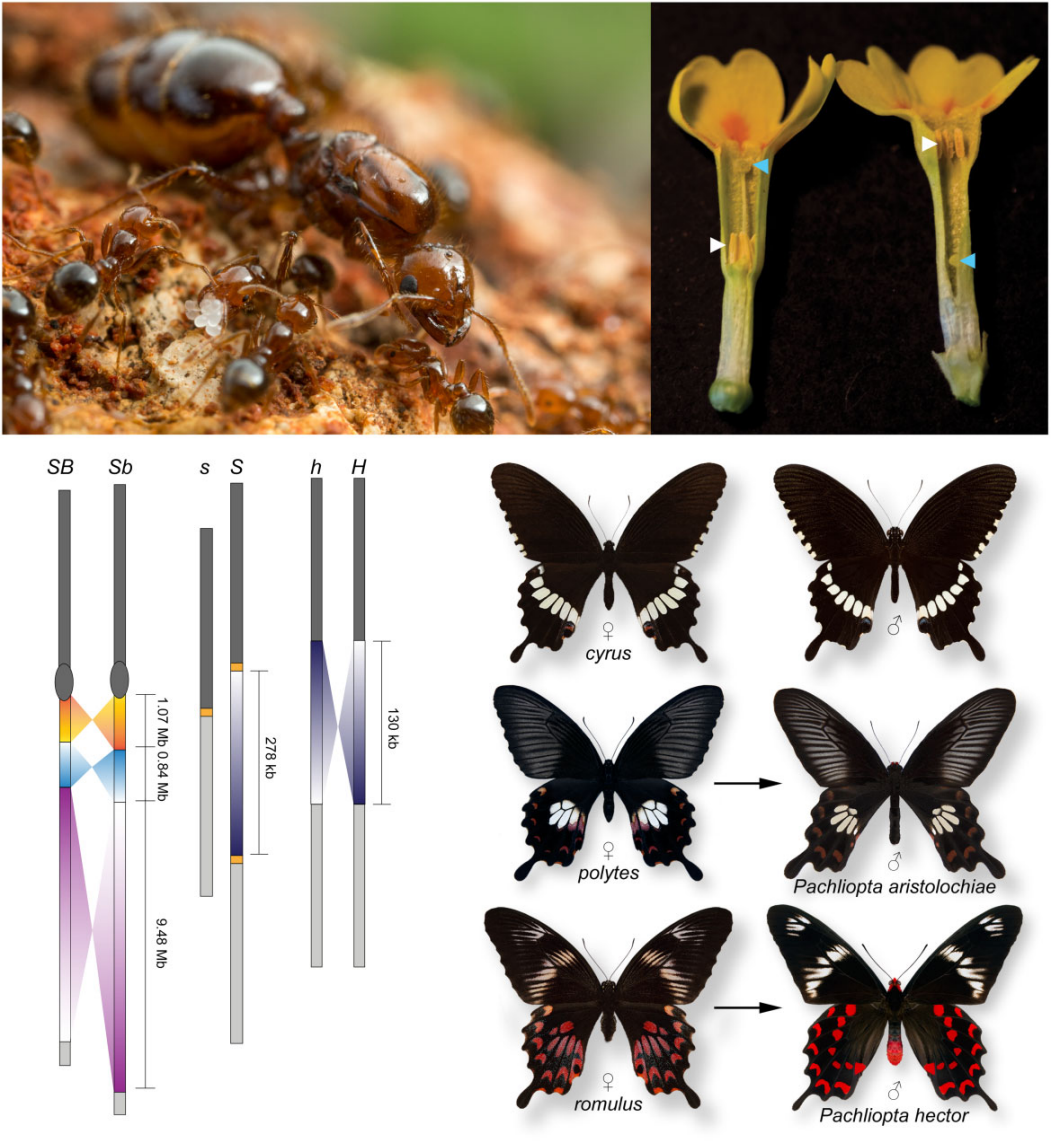
The genomic architectures of supergenes govern colony social form in *Solenopsis invicta* (top left; *SB/Sb* alleles), heterostyly in *Primula veris* (top right; *S/s* alleles), and polymorphic female-limited mimicry in *Papilio polytes* (bottom right; *H/h* alleles). Image courtesy of Tanja Slotte. Photo Credits: Alex Wild (*Solenopsis*), Tanja Slotte (*Primula*), and Krushnamegh Kunte (*Papilio*).

In their review, Slotte and her collaborators discuss the ways in which new sequence data have challenged some classical supergene models (fig. 1). “One aspect that I find really fascinating is how empirical genomic studies are still yielding surprises when it comes to the origin and the genetic architecture of classic supergenes,” says Slotte. An example of this is the case of the supergene governing wing pattern mimicry in the butterfly *Heliconius numata*. *Heliconius numata* exhibits Müllerian mimicry, displaying one of seven different wing patterns, each of which mimics a different local species of the poisonous butterfly Melinaea, thus reinforcing their protection against predators. Recent data show that, rather than arising via the classical model of sequential mutation followed by inversion, the inverted chromosomal arrangement in *H. numata* arose via introgression from another *Heliconius* species. According to Slotte, this is a scenario that should be further explored using simulations and modeling.

Another surprise stemmed from recent studies of the *S*-locus supergene governing heterostyly in primroses. Heterostyly is a common plant adaptation resulting in the presence of two distinct flower morphs (S- and L-morphs) within a species. In each morph, the male and female reproductive organs in the flower are arranged in such a way that it is difficult for an individual plant to fertilize itself, limiting inbreeding and promoting outcrossing. Classical theories posited that S-morph flowers were heterozygotes (carrying two different versions of the *S*-locus supergene), whereas L-morph flowers were homozygotes (carrying two identical versions). Instead, new evidence reveals that primroses with S-morph flowers harbor an insertion spanning five genes that is absent from L-morph primroses, making S-morph plants hemizygotes (carrying a single copy of the *S*-locus).

In their article, Slotte and colleagues further take advantage of advances in bioinformatics and computational biology to reveal new insights into supergene evolution. “When it comes to delineating expected patterns of evolution,” says Slotte, “we benefit enormously from new efficient and flexible simulation software.” Indeed, based on the new findings regarding the primrose *S*-locus supergene, Slotte and her co-authors used SLiM, a forward simulation program, to compare two hypothetical *S*-locus systems: the classic inversion model and the new model, in which an insertion leads to a supergene that is hemizygous in S-morphs. Their results revealed that the inversion accumulated more than six times more deleterious mutations than the hemizygous region and that each of these mutations on average had more deleterious effects, demonstrating that the specific genomic architecture of a supergene has a powerful effect on its ultimate degeneration and evolutionary fate. 

In addition to the insights revealed by new sequence data and simulations, scientists have an unprecedented opportunity to study supergene functionality thanks to new genetic and molecular biology tools. According to Slotte, “Elucidating the function of genes located in supergenes has long been difficult, as by definition it is challenging to fine-map anything in a non-recombining region, but with the help of new genome editing techniques, this is now becoming increasingly feasible.” In particular, Slotte hopes to use a combination of these approaches to study a new supergene target: “I recently got a great opportunity to bring this work to a new level through a Starting Grant from the European Research Council to study the supergene that governs heterostyly in wild flaxseed species (*Linum*). This work is now ongoing, and I am very excited about using this system to study supergene and plant mating system evolution.” 
